# Oxygen Relieves the CO_2_ and Acetate Dependency of *Lactobacillus johnsonii* NCC 533

**DOI:** 10.1371/journal.pone.0057235

**Published:** 2013-02-26

**Authors:** Rosanne Y. Hertzberger, R. David Pridmore, Christof Gysler, Michiel Kleerebezem, M. Joost Teixeira de Mattos

**Affiliations:** 1 Molecular Microbial Physiology, Swammerdam Institute for Life Sciences, University of Amsterdam, Science Park, Amsterdam, The Netherlands; 2 NIZO food research, Ede, The Netherlands; 3 Kluyver Centre for Genomics of Industrial Fermentation, The Netherlands; 4 Nestlé Research Centre, Vers-chez-les-Blanc, Switzerland; 5 Host Microbe Interactomics Group, Wageningen University, Wageningen, The Netherlands; University Medical Center Utrecht, The Netherlands

## Abstract

Oxygen relieves the CO_2_ and acetate dependency of *Lactobacillus johnsonii* NCC 533. The probiotic *Lactobacillus johnsonii* NCC 533 is relatively sensitive to oxidative stress; the presence of oxygen causes a lower biomass yield due to early growth stagnation. We show however that oxygen can also be beneficial to this organism as it relieves the requirement for acetate and CO_2_ during growth. Both on agar- and liquid-media, anaerobic growth of *L. johnsonii* NCC 533 requires CO_2_ supplementation of the gas phase. Switching off the CO_2_ supply induces growth arrest and cell death. The presence of molecular oxygen overcomes the CO_2_ dependency. Analogously, *L. johnsonii* NCC 533 strictly requires media with acetate to sustain anaerobic growth, although supplementation at a level that is 100-fold lower (120 microM) than the concentration in regular growth medium for lactobacilli already suffices for normal growth. Analogous to the CO_2_ requirement, oxygen supply relieves this acetate-dependency for growth. The *L. johnsonii* NCC 533 genome indicates that this organism lacks genes coding for pyruvate formate lyase (PFL) and pyruvate dehydrogenase (PDH), both CO_2_ and acetyl-CoA producing systems. Therefore, C1- and C2- compound production is predicted to largely depend on pyruvate oxidase activity (POX). This proposed role of POX in C2/C1-generation is corroborated by the observation that in a POX deficient mutant of *L. johnsonii* NCC 533, oxygen is not able to overcome acetate dependency nor does it relieve the CO_2_ dependency.

## Introduction

The *Lactobacillus acidophilus* group was recognized early as the most prevalent inhabitant of the vaginal microbiota [1], [2] and also as the pioneer bacteria in the developing intestinal microbiota of neonates [3]. Various strains and species of the acidophilus group are marketed as functional ingredients in probiotic products, associated with health benefits for the consumer. Therefore, understanding of the physiology of members of this group of lactic acid bacteria is of importance both from a medical and an economical point of view.

One of the probiotics belonging to this group is *Lactobacillus johnsonii* NCC 533, whose genome sequence was published in 2004 [4]. Its probiotic functionalities have been explored in detail, including immuno-modulation [5]–[7] and pathogen inhibition [8]. Additionally, its ability to adhere to the epithelial cell was explored [9], [10].

Analogous to many other members of the acidophilus group, *L. johnsonii* can be considered as a highly auxotrophic species lacking the operons for a range of biosynthetic pathways. The genome of *L. johnsonii* NCC 533 lacks genes for the synthesis of vitamins, purines, fatty acids and all amino acids (except for the interconversion of L-asparagine and L-aspartate and the interconversion of L-glutamate to L-glutamine) [4], [11]. As a consequence, *L. johnsonii* has fastidious growth requirements. Noteworthy in the context of applicability, the organism does not grow autonomously on milk [12].

In addition to the above-mentioned auxotrophies, and analogous to many other closely related species, *L. johnsonii* may require a source of acetate for growth. C2-compounds are required in many anabolic reactions and acetate-mediated stimulation of growth has been reported for lactic acid bacteria that exhibit a predominant homolactic metabolism on hexose sugars, such as *Lactobacillus sakei* [13] and *Lactobacillus delbrueckii* [14]. Acetylation of the muramic and glucosamine residues of the peptidoglycan for instance, involves O-acetylation for which a supply of C2 compounds like acetyl-CoA is essential [15].

Heterofermentative lactic acid bacteria have the capacity for acetate production, and are therefore assumed to be independent of exogenous acetate addition. However, growth of a ΔLDH- *Lactococcus lactis* mutant was reported to be stimulated by acetate which it uses for the conversion to ethanol as a means to regenerate NAD^+^ in order to rescue its redox balance [16].

Another well-described growth requirement is CO_2_. *L. johnsonii,* is a so called capnophilic organism, *i.e.* it has a requirement for either gaseous CO_2_ or bicarbonate supplementation for growth, which is a characteristic that is also observed in many other lactic acid bacteria species [17]–[19]. The C-1 source has been proposed to be required for the synthesis of a common intermediate of the pyrimidine and arginine production pathways, carbamoyl-phosphate. In *L. plantarum* carbamoyl-phosphate can be synthesized from glutamine, ATP and bicarbonate involving two enzymes: pyrimidine-regulated CPS-P (encoded by *carAB*) and arginine regulated CPS-A (encoded by *pyrAaAb*) [20]. Two regulators of this pathway, PyrR1 and PyrR2 control expression of the *pyr*-operon in response to pyrimidine and inorganic carbon levels, respectively [21], [22]. The genes of the *pyr*-operon are conserved amongst many lactobacilli, including *L, johnsonii* NCC 533. Homologues of the *arg*FGH genes for arginine biosynthesis are absent, rendering this species auxotrophic for arginine.

The production and consumption of metabolites, like CO_2_ and acetate, are known to stabilize microbial communities. For example, in yoghurt fermentation, *Streptococcus thermophilus* and *L. delbrueckii* show close metabolic relations with the first species providing the second with CO_2_, acetate, folate, and formate. In exchange, the streptococcal species profits from the proteolytic activities of *L. delbrueckii* [23]. Analogously, it can be anticipated that specific nutritional requirements of microbes play an important role in the composition of the human microbiota. In view of both its industrial potential and its niche in the complex microbial environments where these lactobacilli are generally found, such as the gut, understanding the mechanisms that underlie these growth requirements are important.

Growth requirements may be strongly dependent on the growth conditions. For *L. johnsonii* NCC 533 we observed major differences in growth and viability between aerobic and anaerobic conditions, including a significantly higher viability in the presence of molecular oxygen. This is surprising in view of the observation that *L. johnsonii* is known to produce hydrogen peroxide under aerobic conditions, a compound that is generally assumed to be toxic [24]. The study presented here indicates that the anaerobic dependency of *L. johnsonii* for carbon dioxide and acetate is related to its limited flexibility in pyruvate dissipation pathways, which can be overcome by pyruvate oxidase activity in the presence of oxygen, placing this enzyme in a pivotal position in the central metabolism of *L. johnsonii.*

## Materials and Methods

### Strains and Culture Conditions

*Lactobacillus johnsonii* NCC 533 was obtained from the Nestlé Culture Collection. NCC 533 plus NCC 9333, a *pox*-deletion derivative of NCC 533, were routinely cultured in MRS medium at 37°C under anaerobic conditions. Erythromycin was supplemented at 5 µM/ml as required.

### Anopore Growth and Micro-colony Analyses

The Anopore™ method (inoculation, incubation, imaging) was carried out as described before by den Besten et al. [25]. Multiple Anopore™ inorganic membranes (Anodisc™) were placed on MRS-agar plates and four dilutions (10^2^–10^5^) of an overnight MRS culture of *L. johnsonii* NCC 533 were spotted per slide (to ensure that colonies would be physically separated to allow individual quantification). The plates were incubated at 37°C in Oxoid jars that were filled with a defined gas mixture by vacuuming, followed by replacement with the gas mixture of choice, and repeated 3 times at the start of the experiment as well as after every opening of the jar for sampling. Single Anopore™ slides were removed at different time points and transferred to a microscopic slide with a pronarose layer that contained the cell permeable SYTO9 stain and the cell impermeable propidium iodide stain (*bac*light live/dead staining, Molecular Probes, Invitrogen). SYTO9 enters all cells but is replaced by propidium iodide whenever the membrane integrity is compromised.

For every time point and condition between 20 and 147 microcolonies were randomly selected from one slide and imaged directly without the use of a cover slip or immersion oil. Photographs were taken through both red and green filters with a cooled charge-coupled device camera (Princeton Instruments, SARL, Utrecht, The Netherlands) mounted on an Olympus BX-60 fluorescence microscope. A threshold was applied to create a binary image of the image intensity plots and these were superimposed using ImageJ. An example of such an image is shown in Supplementary materials, Figure S1 in which images of several colonies are combined. The colony size and viability were quantified using the ImageJ. The ImageJ plugin ObjectJ was employed to facilitate colony selection.

### Growth in Liquid Media and Physiological Characterization

Cells were inoculated at an OD_600_ of 0.01–0.05 in fresh medium. Growth was monitored in continuously stirred vessels with 400 ml regular MRS medium [26](for the CO_2_ dependency experiments) or a chemically defined medium (in case of the acetate dependency experiments). The latter medium was described for *Lactobacillus reuteri* [27] and has previously been shown to also support growth of *Lactobacillus johnsonii* NCC 533. Batches were sparged with specific gas mixtures varying in CO_2_ and O_2_ content. Cultures were grown at 37°C with constant mixing (ca. 200 rpm) and pH was maintained at pH 6.5 by automatic 4 M NaOH titration. Cell density was determined by measuring the optical density at 600 nm. Growth rate was determined by fitting an exponential trendline through the data points with a minimal R^2^ of 0.99. In cases of very slow or no growth a trendline was fitted through the data points of the first five hours of incubation (for instance where chemically defined medium is inoculated in the absence of acetate and oxygen).

### Construction of the *pox*-deletion Derivative of *L. johnsonii* NCC533

The deletion of the gene LJ1853 encoding a predicted pyruvate oxidase enzyme was achieved as described previously [28] with the exception that the plasmid pDP749 was used. In pDP749 the erythromycin resistance gene in pDP600-Ery has been flanked by direct copies of the yeast 2-micron plasmid FLP recombination target sites to facilitate excision of the erythromycin resistance marker by the FLP recombinase. The 5′ region was amplified from *L. johnsonii* NCC 533 genomic DNA with the primers A (ATATATGAGCTCAGCAAGAACGGCTTCTGC) and B (ATATATGGATCCAGATGCTGCTTCTGGTGC) introducing SacI and BamHI restriction sites, respectively. The amplicon was SacI-BamHI digested and cloned in similarly digested pDP749, yielding an intermediate plasmid. The 3′ region was amplified using the primers C (GTGAACGGCACCAGGACC ) plus D (ATATATGGTACCGAAGCATATATTGGGGTC), the amplicon obtained was PstI-KpnI digested and cloned in similarly digested intermediate plasmid to yield the *POX*-deletion plasmid pDP887. Plasmid pDP887 isolated from *Lactococcus lactis* was used to transform NCC 533 [11]and loop-in/loop-out gene replacement was achieved as described previously [28]. The deletion was confirmed by PCR analysis.

### Organic Acids Measurement by HPLC

Extracellular metabolite concentrations were determined as described previously [29] using HPLC (LKB and Pharmacia, Oregon City, OR, USA) fitted with a REZEX organic acid analysis column (Phenomenex, Torrance, CA, USA) at 45°C and a RI 1530 refractive index detector (Jasco, Easton, MD, USA). The mobile phase consisted of a 7.2 mM H_2_SO_4_ solution. Chromatograms were analysed using AZUR chromatography software (St. Martin D’Heres, France).

## Results

### CO_2_ Dependency of *L. johnsonii* NCC 533 during Aerobic and Anaerobic Microcolony Growth

To study CO_2_ dependency of *L. johnsonii*, we used a high-resolution and quantitative technique by using Anopore™ slides to visualize growth on plates that were placed in jars with a controlled atmosphere. This allowed for the rapid assessment of growth requirements and in combination with time-resolved microscopic inspection at using live/dead staining, enabled the generation of additional data related to the organisms’ physiological state, viability and population heterogeneity [25], [30], [31].

This set-up was employed to evaluate growth and viability during CO_2_ limitation under aerobic and anaerobic conditions. To this end, Anopore™ slides on MRS-agar plates were inoculated with different dilutions of cells and incubated in jars filled with gas-mixtures varying in CO_2_ and O_2_ content. At regular intervals the viability and size of the colonies were determined using a live/dead *bac*light stain as described in Materials & Methods. The sum of the propidium iodide stained pixels and the SYTO9 stained pixels was used to estimate the size of the colony. The fraction of SYTO9 over all stained pixels was used as a relative measure of viability.

**Figure 1:**
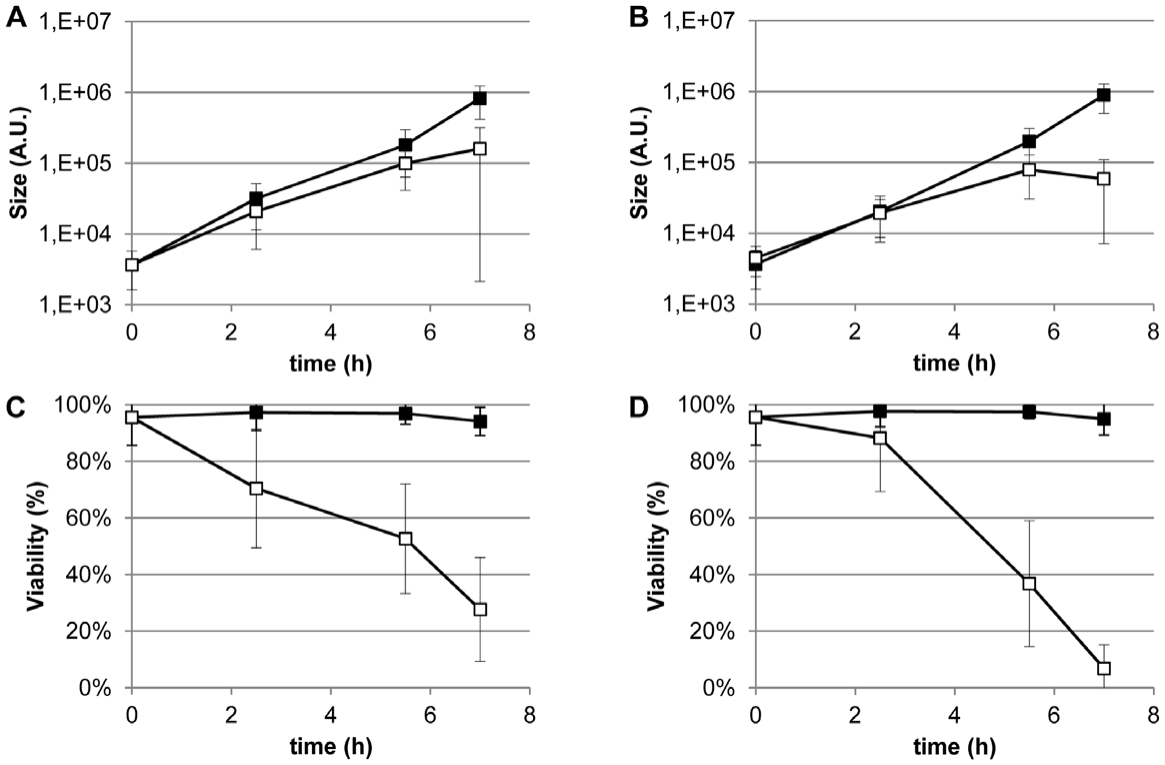
Microcolony growth and viability in environments varying in oxygen and CO_2_ content. *L. johnsonii* NCC 533 is grown on Anopore™ slides that are transferred from a 2 hour pre-incubation period in an N_2_+5% CO_2_ environment to environments that vary in CO_2_ and O_2_ content. Average size of microcolonies grown aerobically (A) and anaerobically (B) and average viability of microcolonies grown aerobically (C) and anaerobically (D). Growth after the pre-incubation was either in the presence (closed symbols) or absence (open symbols) of 5% CO_2_. Data shown are the mean of all colonies counted for that time point and condition ± standard deviation.

CO_2_ supplementation to the gas phase (5%) was found to stimulate growth under both aerobic (air) and anaerobic (N_2_) conditions. When plates were transferred to a CO_2_ depleted environment, growth stagnated after 7 hours, both in aerobic and anaerobic conditions. In the presence of supplemented CO_2_, microcolonies continued growth with an estimated growth rate of 0.79 h^−1^ in the anaerobic, and 0.74 h^−1^ in the aerobic environment, which is comparable to growth rate in liquid culture (data not shown). This growth rate was estimated by fitting an exponential trend line through the average colony size (Figure 1, panels A and B). Growth stagnation was accompanied by loss of membrane integrity observed in microcolonies that are grown without CO_2_ supplementation, whereas microcolonies grown in CO_2_ supplemented environments sustained viability above 90% throughout the experiment (Figure 1, panels C and D).

Notably, microcolonies grown in aerobic atmosphere displayed reduced loss of viability albeit with a higher degree of heterogeneity, as compared to microcolonies grown in a nitrogen atmosphere (Figure 1 C and D). This observation was remarkable since it has been documented that *L. johnsonii* produces hydrogen peroxide in the presence of oxygen [24], which was presumed to reduce growth rate and induce considerable cell death under aerobic conditions. Taken together, these results suggest that CO_2_ depletion leads to loss of membrane integrity and growth stagnation, while oxygenation appears to support extended viability as compared to anaerobic conditions.

### CO_2_ Dependency of *L. johnsonii* NCC 533 during Aerobic and Anaerobic Liquid Growth

**Figure 2:**
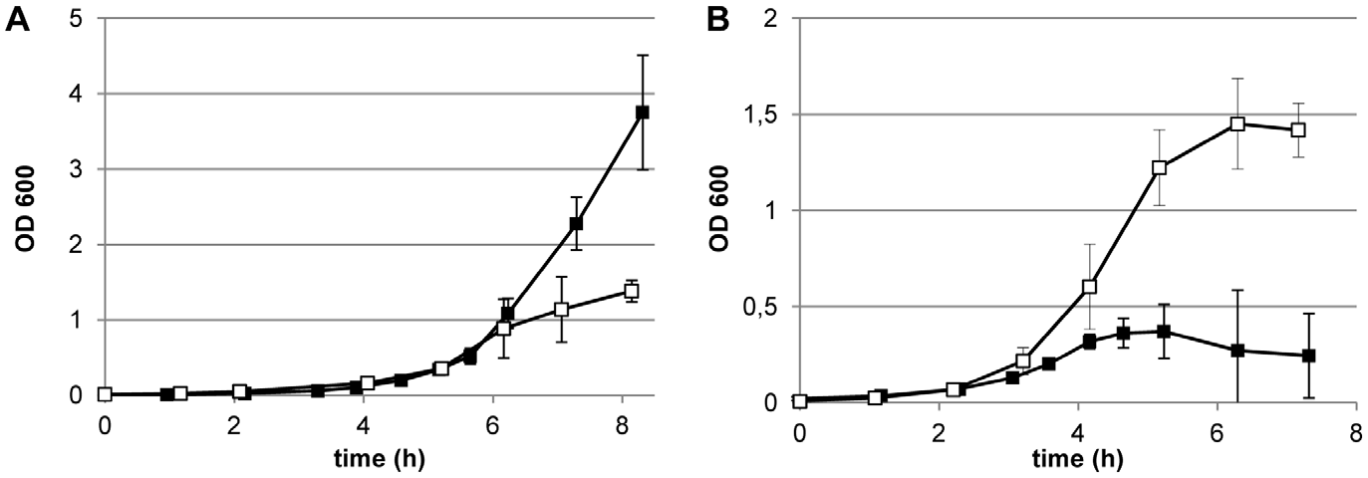
Effect of CO_2_ depletion on aerobic and anaerobic growth. Growth in stirred pH-controlled batch cultures sparged by N_2_+5% CO_2_ (closed symbols) or N_2_+20% O_2_+5% CO_2_ (open symbols) as measured at OD_600_. Data shown are the mean of at least two independent experiments ± standard error of the mean. In panel B, the gas regime was switched after 3 hours of exponential growth from a CO_2_-rich gas to a CO_2_-free gas: N_2_ (closed symbols curve), N_2_+20% O_2_ (open symbols). Growth curves are the average ± standard deviation of triplicate experiments.

To consolidate the results obtained with the Anopore system in the more routinely employed liquid culture conditions, *L. johnsonii* NCC 533 was grown in a pH-controlled stirred batch culture, sparged with predefined gas mixtures at a rate of 750 ml/min. When *L. johnsonii* was grown in MRS medium in this experimental setup, a clear difference between aerobic and anaerobic growth was observed. Anaerobic and aerobic cultures reached an exponential growth rate of 0.85 h^−1^ and 0.69 h^−1^, respectively. After 6 hours of incubation aerobic growth strongly slowed down and eventually the culture entered stationary phase, whereas the anaerobic culture continued growth (Figure 2A). The aerobic growth stagnation was related to the accumulation of H_2_O_2_ in the extracellular growth medium, as is evidenced by the complete prevention of the growth stagnation by the addition of 0.5 mg/ml catalase to the medium (Supplemental material, Figure S2).

To assess the influence of CO_2_ on growth in these conditions, cultures were grown until early-logarithmic phase of growth while sparging a defined gas composition, aerobic (75% N_2_, 20% O_2_ and 5% CO_2_) or anaerobic (95% N_2_ and 5% CO_2_). Subsequently sparging was switched to a CO_2_-free gas mixture. Depletion of CO_2_ in anaerobic cultures resulted in growth stagnation and initiation of cell death within one hour (Figure 2B), whereas in aerobic cultures this effect was not observed and growth continued until it stagnated at a final OD of approximately 1.5, due to the accumulation of H_2_O_2_. Overall, these data show that oxygen supplementation in the gas phase relieves the CO_2_ requirement for growth, both on solid, as well as in liquid media.

### Oxygen Overcomes the Acetate Dependency of *L. johnsonii* NCC 533

**Figure 3:**
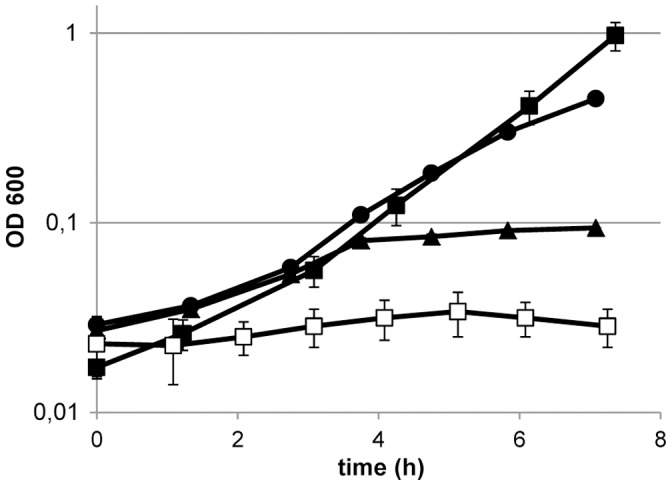
Acetate requirement for anaerobic growth. Growth of *L. johnsonii* NCC 533 in a chemically defined medium with varying concentrations of sodium acetate: 12 mM as in standard CDM (closed square symbols) 120 µM (round symbols), 12 µM (triangular symbols) and without any Na-acetate supplemented (open square symbols) in stirred pH controlled cultures sparged with N_2_+5% CO_2_ at a rate of 500 ml/min. The growth curves are the average of duplicate experiments ± standard error of the mean.

In addition to CO_2_ dependency, growth of many lactobacilli also depends on the presence of acetate in the growth medium [14]. *L. johnsonii* was unable to grow in chemically defined medium without acetate supplementation. Notably, the addition of as little as 12 µM sodium acetate (1/1000 of the regular sodium acetate concentration in the chemically defined medium) allowed for recovery of growth, albeit at a slower rate and yielding lower final biomass concentrations. Acetate supplementation at a 100-fold lower level as compared to its regular concentration in CDM (120 µM) completely restored normal anaerobic growth (Figure 3). These results show that although there is a strict acetate-requirement for growth, this requirement is already fulfilled with concentrations that are substantially below the levels that are normally added to typical *Lactobacillus*-laboratory media, such as MRS or CDM.

**Figure 4:**
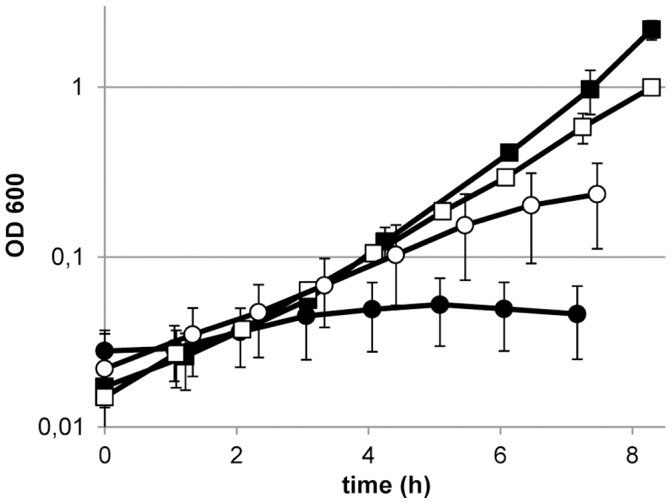
Effect of acetate depletion on aerobic and anaerobic growth. Growth of *L. johnsonii* NCC 533 in a chemically defined medium with 12 mM Na-acetate (square symbols) and without 12 mM Na-acetate (round symbols) in stirred pH controlled cultures sparged with N_2_+5% CO_2_ (closed symbols) or N_2_+20% O_2_+5% CO_2_ (open symbols) at a rate of 500 ml/min. Data are average of independent triplicate experiments ± standard deviation.

To assess whether the acetate requirement of *L. johnsonii* NCC 533 depended on the growth conditions, the strain was grown in chemically defined medium with or without acetate supplementation (12 mM), under aerobic or anaerobic conditions. Analogous to what was observed with respect to the CO_2_ dependency, anaerobic growth of *L. johnsonii* NCC 533 depended more strictly on acetate supplementation as compared to aerobic growth, which could be sustained without an external acetate source, albeit with a slower growth rate and a lower final biomass yield (Figure 4). This implies that the endogenous production of acetate under these conditions may be expected to be in the same range as the 12 µM that allowed similar growth restoration under anaerobic conditions (see above).

Both aerobic and anaerobic growth of *L. johnsonii* in chemically defined medium with 12 mM or 120 µM of acetate were analyzed with respect to acetate metabolism: significant change in extracellular acetate were not detected by HPLC analysis nor by a highly specific and sensitive acetate kinase/pyruvate kinase assay (ref) (results not shown). This result is likely caused by analytical limitations that did not allow detection of the minute amounts of acetate that are required to sustain growth under these conditions, (estimated detection limit in spent medium is 200 µM).

**Figure 5:**
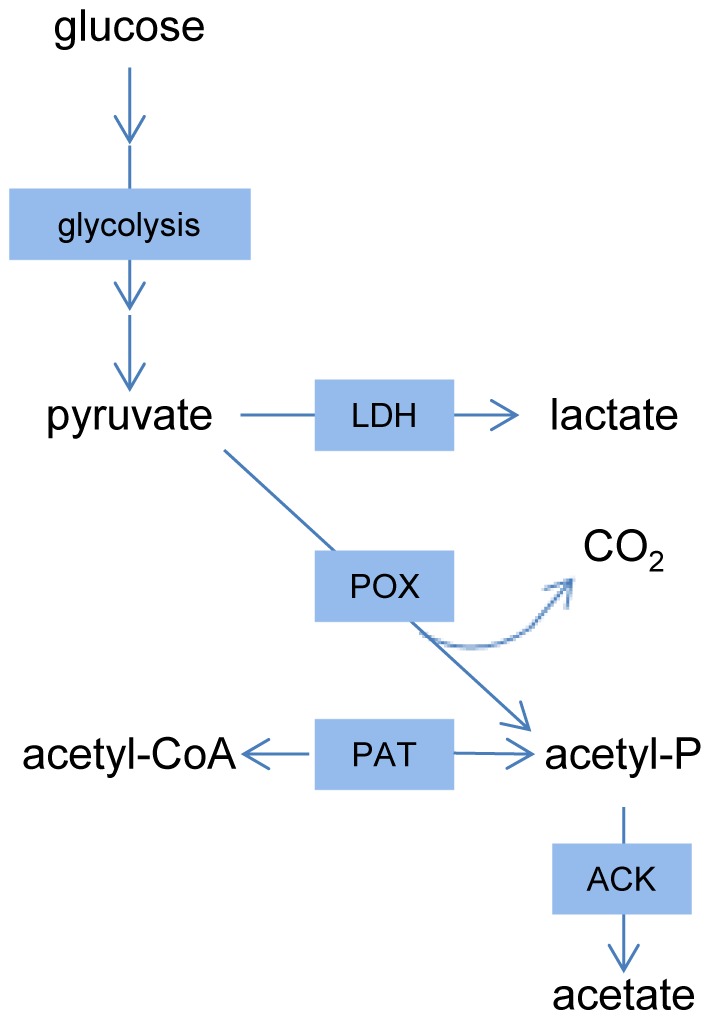
Schematic overview of pyruvate metabolism in *L. johnsonii*. Overview of pyruvate metabolism based on genome annotation. LDH: Lactate dehydrogenase. POX: pyruvate oxidase. ACK: Acetate kinase. PAT: Phosphate acetyltransferase.

In most organisms acetyl-CoA functions as the central C2-intermediate in several biosynthetic pathways. This metabolite can be produced from pyruvate by reactions catalyzed by pyruvate dehydrogenase (PDH) or pyruvate formate lyase (PFL). However, apart from a homologue for one subunit of pyruvate dehydrogenase, the corresponding genes appeared to be absent in the *L. johnsonii* NCC 533 genome [4]. This genotype is shared with the other members of the acidophilus-group (see Supplement, table S1), indicating that these species lack the capacity for autonomous acetyl-CoA production from their central energy metabolism (Figure 5).

The *L. johnsonii* genome does encode an enzyme that could provide the cell with both CO_2_ and acetate, namely pyruvate oxidase (POX). POX catalyzes a reaction that requires molecular oxygen as a co-substrate, and therefore its activity may directly explain the observed physiological consequences of the presence of oxygen (aerobic growth is independent of an external CO_2_ and acetate source). Therefore, we hypothesized that oxygen availability relieves the CO_2_ and acetate dependency by the pyruvate oxidase derived supply of both these metabolites.

### Acetate and CO_2_ Dependency of a *pox*-deletion Mutant

**Figure 6:**
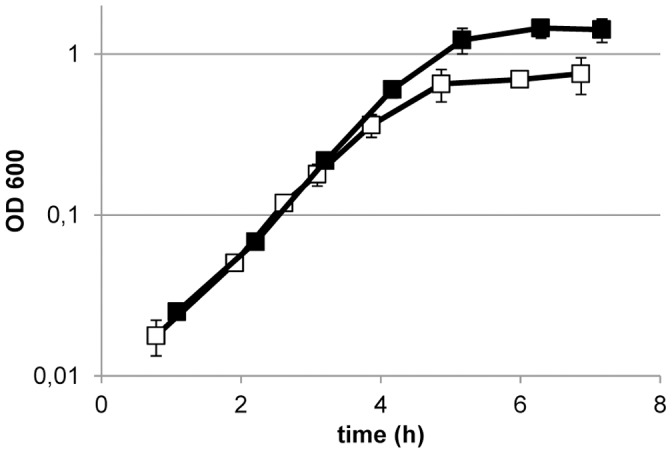
Aerobic CO_2_ requirement of a NCC 9333 mutant. Growth of the NCC 533 (closed symbols) and NCC 9333 (open symbols) as measured at OD_600_ in stirred batch cultures sparged with N_2_+20% O_2_+5% CO_2_. The gas regime was switched after 3 hours of exponential growth to N_2_+20% O_2_. Data are the average of quadruple independent experiments ± standard deviation.

To test the proposed hypothesis, a *pox* deletion derivate that lacks the pyruvate oxidase encoding gene was constructed. Under anaerobic condition in an atmosphere supplemented with 5% CO_2_, the growth rate of the mutant in MRS was similar to that observed for NCC 533. Moreover, under these conditions the wild-type and its *pox*-deletion derivative displayed a comparable growth arrest upon CO_2_ depletion. However, under aerobic conditions, shutting down the 5% CO_2_ supply elicited rapid growth stagnation of the *pox* mutant (Figure 6), which is in clear contrast to the wild-type that continues to grow under these conditions. Clearly, the deletion of *pox* resulted in a *L. johnsonii* mutant that depended on exogenous CO_2_ supplementation for aerobic growth. This fully supports the proposed pivotal role of the *pox*-encoded pyruvate oxidase enzyme in the generation of this essential C1-source under these conditions.

Analogous to the CO_2_ supply provided by the POX-pathway under aerobic conditions, it would be expected that this pathway also provides an acetate supply when oxygen is available. Consequently, the *pox* mutant would be expected to be more hampered aerobically in media that lack exogenous acetate as compared to the wild-type strain.

**Figure 7:**
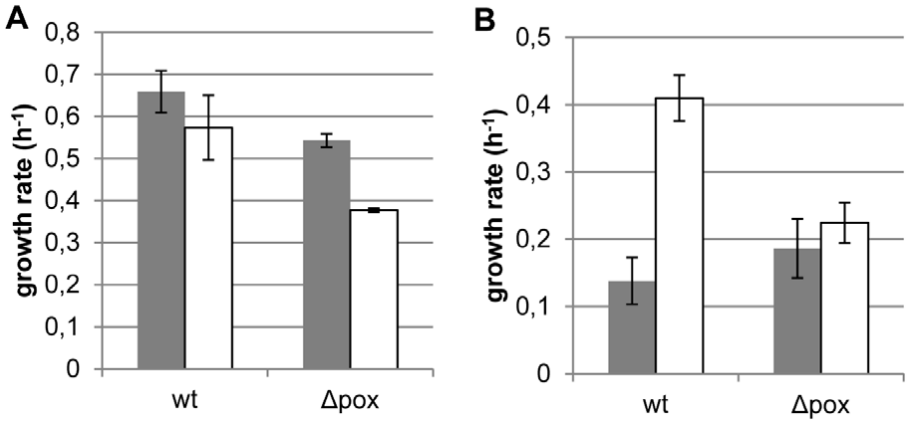
Acetate requirement of a Δpox mutant. Growth rate of *L. johnsonii* NCC 533 in the standard chemically defined medium with (panel A) and without 12 mM Na-acetate (panel B) in stirred pH controlled aerobic batch cultures (open bars) or anaerobic batch cultures (closed bars). Growth rates were determined as explained in Materials & Methods. Data are average of triplicate experiments (panel A) and duplicate experiments (panel B) ± standard error of the mean.

Generally, the pyruvate oxidase deficient mutant displayed slower growth rates than the wild type, independent of the presence of oxygen (Figure 7A). However, growth of the *pox* mutant in the absence of acetate differed considerably, i.e., the typical oxygen relief of the acetate dependency that was observed for the wild type was not observed for the *pox* mutant (Figure 7B), which supports our hypothesized role of pyruvate oxidase in generating C2-compounds.

## Discussion

*Lactobacillus johnsonii* is generally described as an anaerobic fastidious lactic acid bacterium. Fastidious because its growth is dependent on supplementation of various nutrients to its growth medium, and anaerobic because oxygen cannot be used for respiration. Moreover, *L. johnsonii* produces hydrogen peroxide when grown under aerobic conditions, which inhibits growth. Here we present an example that auxotrophy can be dependent on external conditions that seemingly are not related to the nutrient requirement: we show that anaerobicity actually exacerbates the fastidious nature of *L. johnsonii* NCC 533 since the presence of oxygen is shown to relieve at least two of its anaerobic growth requirements, i.e., the requirement for acetate and CO_2_.

Both on plates and in liquid culture, *L. johnsonii* showed clear CO_2_ dependent growth. However, the oxygen relief of this dependency was more apparent in liquid culture than on solid medium, as illustrated by the observation that aerobic growth on plates without CO_2_ still resulted in smaller colonies and reduced viability. In contrast, these CO_2_ dependent phenotypic differences were completely abolished by oxygen supplementation in liquid culture. One explanation for the observed difference could be found in the ambient pH, which is controlled at 6.5 in liquid culture and is uncontrolled in the Anopore experiment. It should be noted in this context that pH influences the equilibrium between the different dissolved carbonic species; CO_2_ dissolves in water as H_2_CO_3_ (pK_a_ 6.1) and the latter species may be deprotonated in a pH dependent manner to generate HCO_3_^−^ and CO_3_^2−^, respectively. Thus, lower pH values shift the equilibrium resulting in release of CO_2_ from the solution to the effect that less CO_2_ is available to the bacteria.It is to be expected that on solid media especially the local pH within the direct environment of emerging microcolonies drops substantially below 6.1 due to lactic acid production. These micro-scale differences in environmental conditions experienced by bacteria grown in microcolonies versus liquid cultures may explain the observed CO_2_ dependency differences observed.

Like the other species in the acidophilus-group (*L. delbrueckii, L. gasseri, L. johnsonii, L. crispatus, L. amylovorus, L. helveticus*), the genome of *L. johnsonii* lacks two major systems for the production of C2- and C1-compounds, namely the pyruvate dehydrogenase complex (PDH) and pyruvate-formate lyase (PFL) producing acetyl –CoA (Supplemental material, table S1). Instead, the genomes of these species all encode the pyruvate oxidase gene that can provide a metabolic source of C2-compounds whenever molecular oxygen is available for the POX reaction. The primary habitat of *L. johnsonii* is considered to be the intestine, which is a predominantly anaerobic environment and would therefore not support POX mediated C2-production. However, in close vicinity to the mucosal tissues, local and a steep oxygen gradient may be encountered [32] that may allow for the POX-mediated contribution to metabolism. Notably, preliminary transcriptome studies of *L. johnsonii* grown under anaerobic, aerobic and CO_2_ depleted conditions did not reveal regulation of the *pox* gene expression, suggesting that the enzyme is constitutively expressed. Based on the physiological observations both on plate and in liquid culture, combined with the absence of these genes, we hypothesized that pyruvate oxidase activity would play a pivotal role in the acetate and CO_2_ supply for the cell. Indeed, a *pox*-deletion derivative of *L. johnsonii* did not display a higher growth rate under aerobic conditions in the absence of acetate, such as observed in the wild type strain. Moreover, whereas the wild type strain continued to grow upon a switch to CO_2_ depletion, growth of the mutant stagnated at a lower biomass concentration. The observed time lapse between the onset of flushing with CO_2_ free gas and the actual CO_2_ depletion of the system is most likely due to the slow removal of all carbonic species at a pH higher than 6.1 (the pK_a_ of carbonic acid). Both results show that, in contrast to the wild type, the *pox*-mutant has lost the ability to aerobically generate CO_2_ and acetate. This corroborates the proposed role of pyruvate oxidase in the generation of C1 and C2 metabolic intermediates.

It was observed that the *pox* mutant has a lower growth rate, both aerobically and aerobically. Although it can be argued that under aerobic conditions the pox gene might play a role in protection against its reaction product, hydrogen peroxide by allowing for a faster production rate of ATP via the production of acetyl-phosphate and subsequent generation of ATP by acetate kinase [33], this argument does not hold for anaerobic growth conditions. So far, no specific role for POX under these conditions can be brought forward and the cause of the effect of the deletion on growth remains to be elucidated.

The major dependency of *L. johnsonii* on pyruvate oxidase for the supply of these compounds was rather unforeseen since many other pathways are known and present in *L. johnsonii* that can render CO_2_ and acetate. Phosphoketolase, for instance, catalyzes the deacetylation of xylulose-5-phosphate which yields acetyl-phosphate. Similarly, CO_2_ can be produced through decarboxylation of amino acids, oxaloacetic acid and phosphopantotenoyl. However, acetate and CO_2_ are both required for growth of *L. johnsonii* in the absence of oxygen, even though very low concentrations of acetate (<120µM) already suffice for growth. This suggests that the flux through these pathways compared to pyruvate oxidase is marginal.

It is uncertain, however, that the lactobacilli that do possess PDH and PFL encoding genes (Supplemental materials, Table S1), can actually employ these pathways for the synthesis of C1 and C2-compounds under aerobic conditions. Literature suggests that *L. plantarum* does not possess a functional pyruvate dehydrogenase pathway, since acetate production does not require CoA and is not hampered by PDH-inhibitors like arsenate [34], [35]. In addition, pyruvate formate lyase activity has been reported to be highly oxygen sensitive and is only considered active under anaerobic conditions [36]. The presence of genes predicted to encode PFL or genes that resemble the PDH-genes of other organisms does not preclude that a species still depends on pyruvate oxidase under aerobic conditions for the production of C2 and C1 components, analogous to what we concluded for *L. johnsonii*.

Clear data to support this hypothesis are lacking, although CO_2_ dependency of *L. plantarum* was also reported to cause a characteristic growth stagnation under aerobic conditions [19]. In addition, another study showed that a pyruvate-oxidase deficient mutant of *L. plantarum* is hampered in its acetate production capacity [37], [38], supporting the role of this enzyme in aerobic acetate supply in lactobacilli that have a broader genetic arsenal.

The effect of deletion of *pox* in *L. johnsonii* confirms the role of POX in the generation of both C1 and C2 sources (CO_2_ and acetate) required for growth. However, a byproduct of pyruvate oxidation by POX is hydrogen peroxide, of which the accumulation induces oxidative stress that leads to premature growth arrest under aerobic conditions [24]. This brings us to the intriguing conclusion that oxygen appears to both benefit and harm *L. johnsonii*. Under aerobic conditions, clearly, a lower biomass yield is reached (Supplemental material, Figure S1) on the one hand, presumably as a consequence of hydrogen peroxide production. On the other, our data also establish clearly that oxygen can increase the metabolic capacity of the strain, relieving some of its fastidious growth requirements. These opposing consequences of oxygen presence suggest that a micro-aerobic environment may be optimal for growth of *L. johnsonii* NCC 533.

Here we have refined the metabolic requirements of *L. johnsonii* NCC 533 and pinpointed the pivotal role of the *pox* gene in the requirement for C1 and C2 sources. These findings can provide novel clues for the optimization of growth conditions of these commercially relevant microbes, and may in more general terms facilitate a more efficient regime for the production of probiotics belonging to this group of lactobacilli.

## Supporting Information

Figure S1 **Effect of catalase on aerobic growth.** Figure 1: Growth of *L. johnsonii* NCC 533 in MRS medium supplemented with 0.5 mg/ml catalase (open symbols) and regular MRS medium (closed symbols) in either static tubes with limited headspace (round symbols) or in shake flasks (square symbols). Depicted are the averages of duplicate experiments ± standard error of the mean.
(TIF)

Figure S2 **Superimposed image of** ***bac*** **light-stained microcolonies.** Composite picture in which images of colonies after 7 hours of growth in environments that vary in oxygen and CO_2_ content are grouped. Images were thresholded, colors were assigned artificially and superimposed as described in Materials & Methods.
(TIF)

Table S1 **Presence of genes for pyruvate dehydrogenase or pyruvate formate lyase in** ***Lactobacilli*** **.** Overview of pyruvate dehydrogenase and pyruvate formate lyase encoding gene prevalence in lactobacilli (Table A) and in species belonging to the *Lactobacillus acidophilus* group (Table 1B). If no gene was found, a BLAST search was performed using the protein sequence of the homologue in *L. plantarum* WCFS1. Shown are the query coverage and the e-value.
(DOCX)
