# Supplementary figures and images for: Oxygen Relieves the CO2 and Acetate Dependency of Lactobacillus johnsonii NCC 533
Source: PLoS One. 2013 Feb 26;8(2):e57235. doi: 10.1371/journal.pone.0057235 (PMC3582564; doi:10.1371/journal.pone.0057235)

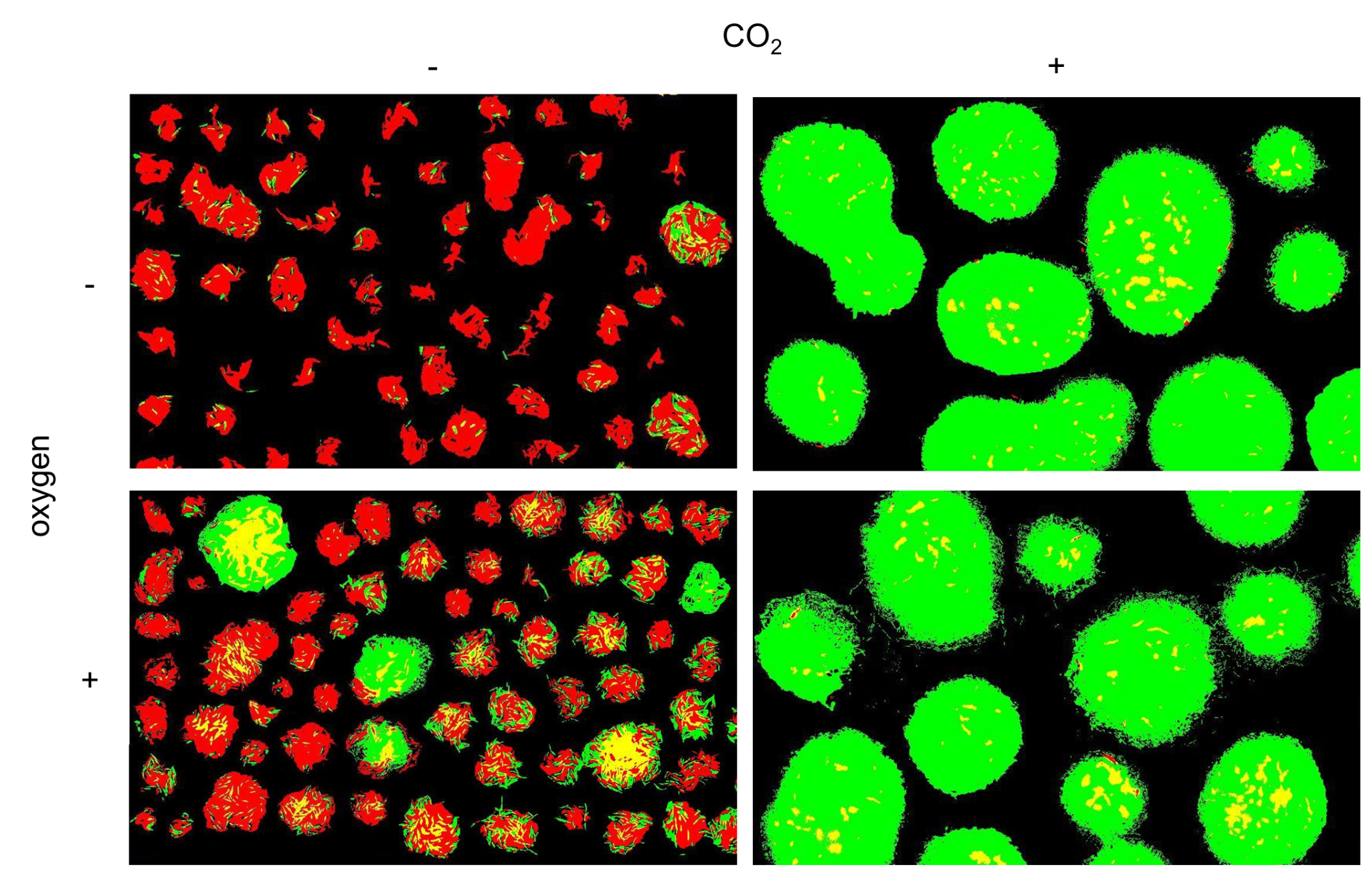

Supplement: Figure S1 — Effect of catalase on aerobic growth. Figure 1: Growth of L. johnsonii NCC 533 in MRS medium supplemented with 0.5 mg/ml catalase (open symbols) and regular MRS medium (closed symbols) in either static tubes with limited headspace (round symbols) or in shake flasks (square symbols). Depicted are the averages of duplicate experiments ± standard error of the mean. (TIF) [file pone.0057235.s001.tif]

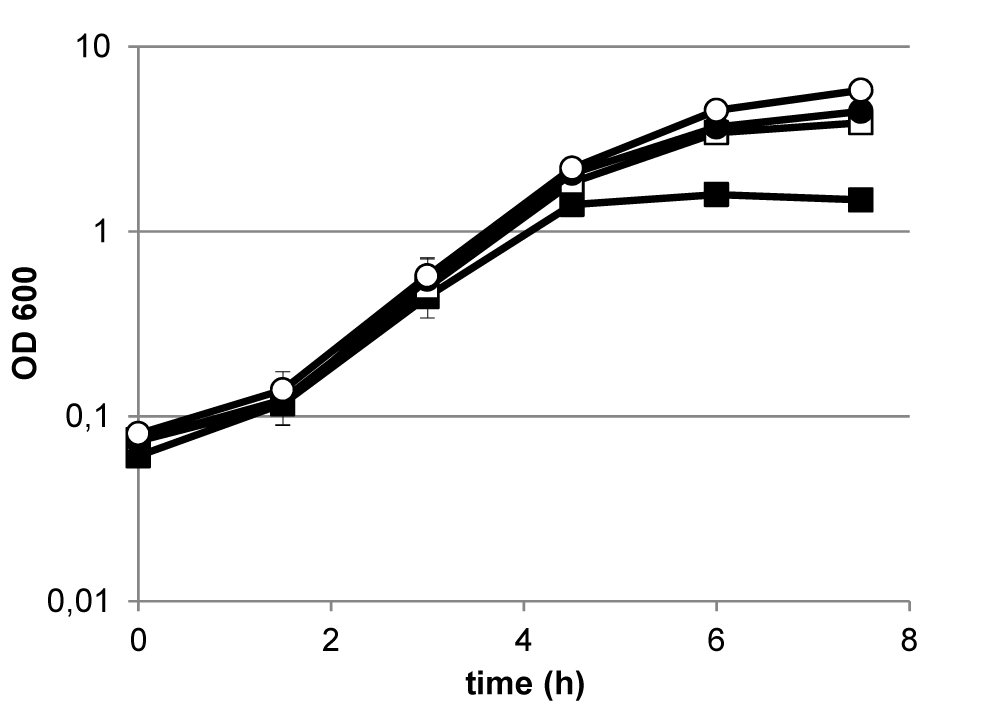

Supplement: Figure S2 — Superimposed image of bac light-stained microcolonies. Composite picture in which images of colonies after 7 hours of growth in environments that vary in oxygen and CO2 content are grouped. Images were thresholded, colors were assigned artificially and superimposed as described in Materials & Methods. (TIF) [file pone.0057235.s002.tif]
