# Supplementary material for: Oxygen Relieves the CO2 and Acetate Dependency of Lactobacillus johnsonii NCC 533
Source: PLoS One. 2013 Feb 26;8(2):e57235. doi: 10.1371/journal.pone.0057235 (PMC3582564; doi:10.1371/journal.pone.0057235)
Supplement: Table S1 — Presence of genes for pyruvate dehydrogenase or pyruvate formate lyase in Lactobacilli . Overview of pyruvate dehydrogenase and pyruvate formate lyase encoding gene prevalence in lactobacilli (Table A) and in species belonging to the Lactobacillus acidophilus group (Table 1B). If no gene was found, a BLAST search was performed using the protein sequence of the homologue in L. plantarum WCFS1. Shown are the query coverage and the e-value. (DOCX) [file pone.0057235.s003.docx]

| **Supplementary Table S1A** | | | | | | | | |
| --- | --- | --- | --- | --- | --- | --- | --- | --- |
|  | PDH complex | | | | PFL | | | |
| *Lactobacillus* | *pdhA* | *pdhB* | *pdhC* | *pdhD* | *pflA* | *pflB* | *pflE* | *pflF* |
| *plantarum* WCFS1 | lp_2154 | lp_2153 | lp_2152 | lp_2151 | lp_3314 | lp_3313 | lp_2596 | lp_2598 |
| *salivarius* UCC118 | lsl_0153 | lsl_0154 | lsl_0155 | lsl_0156 | lsl_1872 | lsl_1873 | - | - |
| *sakei* subsp*. Sakei* 23K | lca_1085 | lca_1084 | lca_1083 | lca_1082 | lca_0973 | lca_0974 | lca_0973 | lca_0974 (66%, 1e^-54^) |
| *rhamnosus* GG | lrhm_1266 | lrhm_1267 | lrhm_1268 | lrhm_1269 | lhrm_1366 | lhrm_1365 | - | - |
| *casei* ATCC 334 | lsei_1305 | lsei_1306 | lsei_1307 | lsei_1308 | lsei_1412 | lsei_1410 | - | - |
| *reuteri* DSM 20016 | lar_0608 | lar_0609 | lar_0610 | lar_0611 | - | - | - | - |
| *brevis* ATCC 367 | lvis_1410 | lvis_1409 | lvis_1408 | lvis_1407 | - | - | - | - |

| **Supplementary Table S1B - *Lactobacillus acidophilus* group** | | | | | | | | |
| --- | --- | --- | --- | --- | --- | --- | --- | --- |
|  | PDH complex |  |  |  | PFL |  |  |  |
| *Lactobacillus* | *pdhA* | *pdhB* | *pdhC* | *pdhD* | *pflA* | *pflB* | *pflE* | *pflF* |
| *johnsonii* NCC 533 | - | lj_1267 (88% 3e^-17^)^1^ | - | lj_1757 (95% 2e^-61^)^2^ | - | - | - | - |
| *acidophilus* NCFM | - | lba_1490 (87% 1e^21^)^1^ | - | lba_1220 (96% 1e^-65^)^2^ | - | - | - | - |
| *helveticus* DPC 4571 | - | - | - | lhv_1961, (94%, 3e^-66^)^2^ | - | - | - | - |
| *delbrueckii* ATCC 11842 | - | - | - | ldb_0759 (95% 3e^-66^)^2^ | - | - | - | - |
| *gasseri* ATCC 33323 | - | - | - | lgas_1554 (95% 3e^-61^) ^2^ | - | - | - | - |

1 BLAST homology to a transketolase

2 Blast homology to a pyrimidine-dinucleotide oxidoreductase
